# Research Status and Development Trend of MEMS Switches: A Review

**DOI:** 10.3390/mi11070694

**Published:** 2020-07-17

**Authors:** Tongtong Cao, Tengjiang Hu, Yulong Zhao

**Affiliations:** State Key Laboratory for Manufacturing System Engineering, Xi’an Jiaotong University, Xi’an 710049, China; tammy0326@stu.xjtu.edu.cn

**Keywords:** MEMS switches, driving principle, reliability, bistable mechanism

## Abstract

MEMS switch is a movable device manufactured by means of semiconductor technology, possessing many incomparable advantages such as a small volume, low power consumption, high integration, etc. This paper reviews recent research of MEMS switches, pointing out the important performance indexes and systematically summarizing the classification according to driving principles. Then, a comparative study of current MEMS switches stressing their strengths and drawbacks is presented, based on performance requirements such as driven voltage, power consumption, and reliability. The efforts of teams to optimize MEMS switches are introduced and the applications of switches with different driving principles are also briefly reviewed. Furthermore, the development trend of MEMS switch and the research gaps are discussed. Finally, a summary and forecast about MEMS switches is given with the aim of providing a reference for future research in this domain.

## 1. Introduction

Switches are essentially used to control the on–off state of circuits and are required to react quickly and accurately to signals. The MEMS switch device is a tiny movable element with three-dimensional structure fabricated by semiconductor technology. MEMS switches offer much lower power consumption, much better isolation, and lower insertion loss compared to conventional field-effect transistors and p-i-n diode switches [1,2,3], and they possess advantages such as small size and high integration. The rise of MEMS switches provides strong technical support for the development of signal control systems. At present, the demand for MEMS switches mainly comes from military security systems [4,5,6,7], the automobile industry [8,9], the wireless communication field [10,11,12,13,14,15], medical apparatus and instruments [9,16], micro-optical electromechanical systems (MOEMS) [17,18,19] and more. Over the last few decades, various types of MEMS switches have been developed. To be familiar with the working mechanism and optimization direction of existing MEMS switches is of great significance for the development of innovative MEMS switches. However, there is a lack of a comprehensive classification of MEMS switches.

MEMS switches can be classified in a variety of ways [20], such as according to whether there is an additional driving source, and the existing MEMS switches can be segmented into passive MEMS switches and active MEMS switches. Passive MEMS switches exploit their own system to induce changes and absorb energy for inertial actuation [21,22]. This driving principle has better long-term storage performance and resistance to electromagnetic interference owing to no need for extra energy [23]. Active drive refers to the use of external energy to drive movable electrodes to change the on–off state of switches. The drive of MEMS switches involves magnetic energy, electrical energy, photochemical energy and other energy fields, which are converted into mechanical energy to generate displacement [24,25].

MEMS switches can be roughly divided into silicon-based MEMS switches and non-silicon-based MEMS switches according to the different processing materials. Silicon-based MEMS switches are usually fabricated on SOI (silicon-on-insulator) wafers with the advantages of high shape precision and a simple process [26,27]. However, if the structural layer material of the silicon-based switch is used directly for contacts, the contact resistance will be too large compared with conductor materials, resulting in an unobvious signal. To reduce the contact resistance, it is necessary to apply a layer of low-resistivity metal on the contact surface of the electrodes [28]. In addition, silicon is not suitable for high impact and high load applications either as a structure layer or as a substrate [29]. On the other hand, non-silicon switches are mainly fabricated from LIGA (lithographie, galvanoformung and abformung) or ultra-precision processing technology. For metal-based switches, multi-layer suspended movable structures are usually fabricated from Ni via micro-electroplating [30,31,32]. In contrast to the properties of silicon-based switches, metal structures provide excellent electrical conductivity, as well as good mechanical properties and toughness. Although this switch solves the problem of high contact resistance, the maturity of metal microstructures manufacturing is relatively low. During processing, the structure is prong to deformation [33], leading to a low yield.

What is more, according to the contact modes, MEMS switches can be grouped into resistive switches and capacitive switches, a classification quite common seen in RF (radio frequency) MEMS applications. Capacitive switches are turned on or off through capacitance coupling [34], and these types of switches are suitable for high-frequency (about 3 MHz to 30 MHz) applications [35,36]. On the other hand, resistive switches are generally used in the lower frequency band (about 30 KHz to 300 KHz) of the radio frequency signal [37]. Low contact resistance, usually less than 1–2 ohms, is one of the important performance requirements of MEMS switches [38].

Of course, MEMS switches can be divided into laterally actuated switches [13,39,40,41,42] and vertically actuated switches [43,44,45] The displacement of vertically actuated switches is out-of-plane while that of laterally actuated switches is in-plane.

This review aims to provide detailed insights into the structural design and performance optimization of MEMS switches, based on the literature of the last 20 years. In the second part, the key performance indexes of MEMES switches, especially the influencing factors of reliability, are pointed out. The third part, as the main body of the paper, introduces in detail the different principles of switches and the targeted performance optimization from the aspect of structure. Thereinto, bistable mechanism is used in almost every actuation as an effective method to enhance the contact effect and improve the switching speed. In the design of active switch, there is also the problem of how to realize the insulation between drive signal and switch signal, which has been also mentioned in each section. Each switch has its pros and cons, so designers have made specific improvements to the switches after trade-offs or analyzing the application requirements. Furthermore, the general development trend of MEMS switches is predicted. This review serves the purpose of providing researchers in this field with a reference source.

## 2. Performance Indicators of MEMS Switches

In the development of MEMS switches, their performance is constantly optimized. The key performance indicators of MEMS switches are driving voltage, switching time, power consumption, reliability and so on. Among them, the reliability of MEMS switches is a factor that must be considered in performance design. The neglect of reliability is a major obstacle to the ultimate commercialization of switches. In order to improve the reliability of switches, possible failure modes of switches should be analyzed first. Table 1 below is an analysis of common failure modes of MEMS switches.

To analyze the failure of MEMS switches in detail, capacitive switches and resistive switches should be considered separately. The main problem affecting the reliability of capacitive switches is not the mechanical properties, but the charging issue. The rate of the C/V curve [46] and the stretched exponential for charging [47] can be used to evaluate the failure time of capacitor switches. Goldsmith [48] proposed an efficient accelerated life test method, where a continuous electrical signal is applied to the switch and detects the modulation signal generated by the switch action. The reason for this failure is assumed to be the continuous accumulation of electric charge in the dielectric layer, which eventually leads to the driving voltage drift or latch-up effect. The optimization of capacitive switches should solve the problem of charge accumulation [49]. On the one hand, it can be improved by optimizing the dielectric material such as the dielectric layer material with high dielectric coefficient and low trap density [48,49,50,51,52]. A dielectric-less switch has proved to be an effective method [53]. On the other hand, the voltage can be optimized, such as using high voltage to drive the switch to close and low voltage to maintain the closed state [54], or using bipolar control voltage [55].

The failure of the resistive switches is due to contact fatigue. Mechanical stress causes deformation and wear of contact surfaces, while electrical stress mainly causes electromigration and melting of contact surfaces. Their combined action eventually leads to increased contact resistance or adhesion. Therefore, the choice of contact material is the key to the reliability of the switch, considering such factors as hardness, resistivity, melting point and sensitivity to organic pollutants [56,57]. Soft metals, such as gold, are suitable for reducing contact resistance, but their contact surfaces are prone to microwelding. Ke et al. [58] coated Au contacts with Ru to investigate placing harder materials on top of softer materials for a lifetime enhancement. Yang et al. [59] showed Au–Ni alloy contacts resist material transfer better than Au–Au contacts. In exchange, alloying Au with other metals also results in an increased resistivity. Yaglioglu et al. [60] examined the electrical contact properties of carbon nanotube (CNT)-coated surfaces. The high Young’s modulus and potential for low resistance of CNTs makes them suitable candidates for micro-switch contacts. Experiments have shown that adding a small amount of Pd or Pt to the gold increases the lifetime of the device, but the contact resistance increases only a small amount [38]. In order to prevent the degradation of switch contact, apart from preventing the mechanical damage of the contact surface, it is necessary to improve the sealing of the packaging to prevent organic or inorganic pollution [61].

Both residual stress and temperature affect the switching capabilities of MEMS switches. This means that changes in operating temperature or increases in residual stress may increase the actuation voltage of MEMS switches. The actual driving voltage of the switch is often very different from the design value. One of the reasons is the influence of residual stress. The residual stress in the movable structure will accelerate the fatigue and reduce the durability of MEMS switches [62]. Residual stress formed during micro-machining is the main factor that affects the reliability of MEMS switches [63,64]. Thermal residual stress is generated during the thermal loading-unload cycles during the plasma etching stage. In surface micromachining process, multilayer metals are deposited on the substrate. The difference between the thermal expansion coefficient of different material layers leads to the formation of residual stress. It affects the flatness of the fabricated switch, thus affecting the static and dynamic characteristics of the switch. For example, the compressive stress increases the pull-in voltage and reduces the switching time [65]. Temperature is the most common failure acceleration factor. The experimental results show that the change in temperature accelerates the failure modes, such as charge capture, mechanical creep and contact degradation [66].

The key performance of the switch, such as driving voltage and switching time, is closely related to the driving principles. Therefore, it is necessary to introduce the principles and optimization performance structurally of each driving mode, respectively and in detail.

## 3. Classification of MEMS Switches Based on Driving Principles

According to the driving principles [67], MEMS mechanical switches can be roughly divided into passive inertial switches, electrostatic switches, electro-thermal switches, electromagnetic switches, piezoelectric switches and shape memory alloy switches [68], etc. Table 2 provides a summary of the key performance comparison of several mainstream MEMS mechanical switches.

### 3.1. Passive Inertial Switches

MEMS inertial switches are special acceleration sensors used to detect the threshold acceleration [69]. The microinertial switch based on MEMS technique is generally designed with a flat plate structure, which is characterized by miniaturization, high reliability and a low cost.

**Figure 1:**
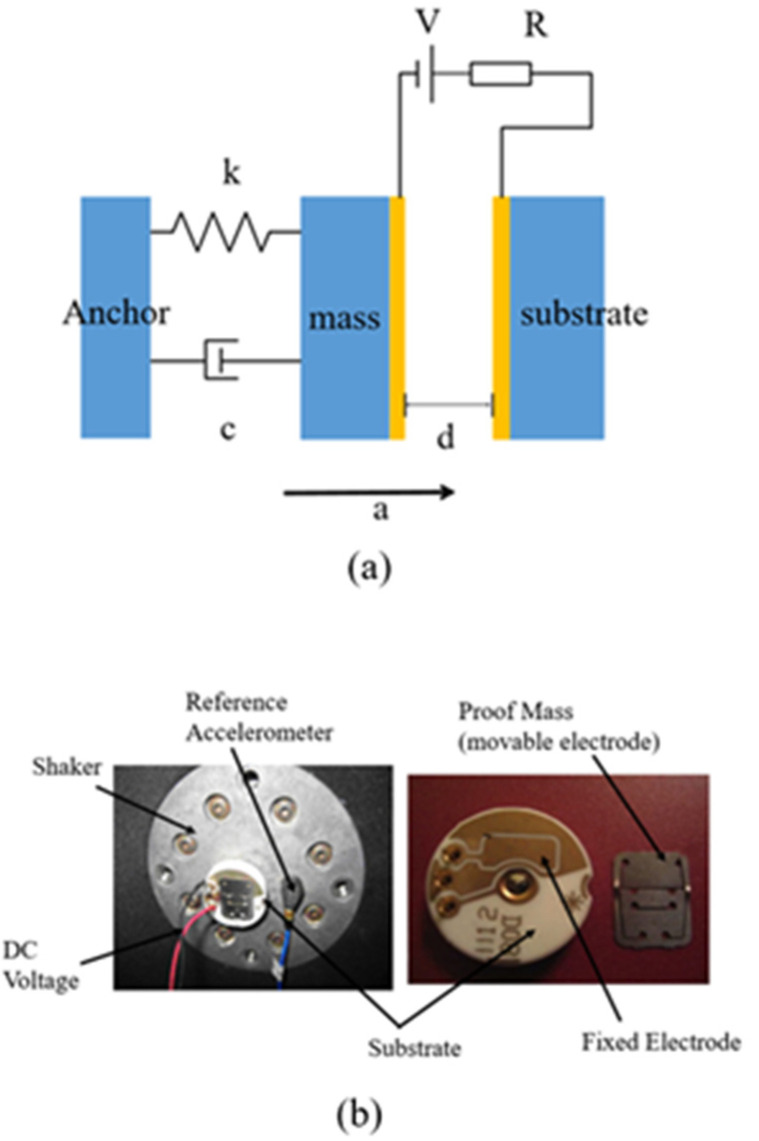
(**a**) The spring-mass -damping system model of inertial switches; (**b**) pictures of a capacitive accelerometer fabricated and its experimental setup built by Younis (2007 [70]).

Under the premise that the gas damping and structural damping cannot be ignored in the dynamic response, the basic model of inertial switches can be simplified as a spring-mass -damping system, as shown in Figure 1a. When the acceleration applied in the sensitive direction of the switch is at or above the threshold level, the movable electrode moves along the sensitive direction until the relative displacement reaches the distance *d* between the two electrodes, and the movable electrode contacts with the fixed electrodes to turn the switch on.

In this process, the equation of motion of the mass block can be described as:

(1) $$m\ddot{x}+c\dot{x}+kx=ma$$

where *m*, *c*, and *k* are the weight of the proof mass, the damping coefficient and the elasticity coefficient of the movable electrode, respectively. *x* is the relative displacement between the moving electrode and the fixed electrode, and *a* represents the acceleration exerted by the outside world on the switch. Inertial switches are discussed below from the aspects of acceleration threshold and contact effect.

Most inertial switches are passive devices, but sometimes switches are designed to be active in order to regulate the threshold. Younis et al. [70] tested two commercial capacitive inertial switches fabricated by Sentasa Technologies [71] (see Figure 1b). The test results showed that the acceleration threshold is linear with the DC voltage for the tunable threshold-acceleration switch. Besides meeting the function of tunable threshold, this kind of switches also have shortcomings: increased volume and power consumption due to added power supply and vulnerability to external electromagnetic interference. Therefore, the passive acceleration switch still plays an irreplaceable role in some applications.

For different application requirements, uniaxial switches [72,73,74,75], biaxial switches [76,77,78], tri-axial switches [79,80] gradually appeared. In the development of MEMS switches, not only the number of acceleration directions have been expanded, but also the axial sensitivity of the switch has been improved [80]. For instance, Currano et al. [81] proposed a triaxial inertial switch based on the symmetrical spiral springs, in which five switches are integrated. In 2014, Chen et al. [80,82] designed and fabricated an all-metal triaxial inertia switch. A triaxial inertial switch can be used instead of multiple uniaxial inertial switches to monitor acceleration in multiple directions and avoid complex installations.

**Figure 2:**
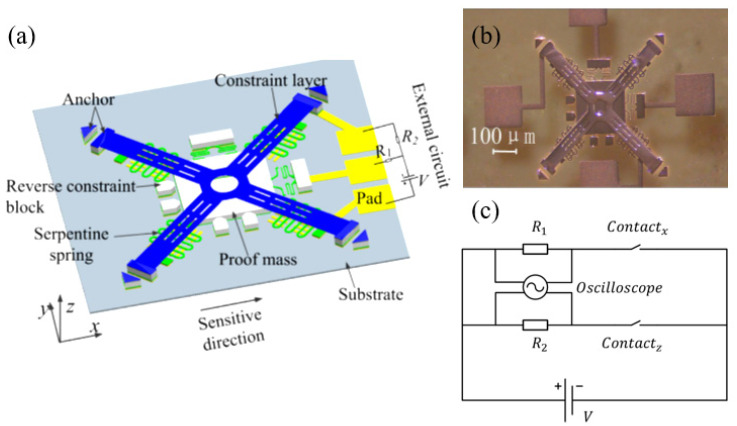
The tri-axial MEMS inertial switch with shock-resistibility (Xu 2016 [83]): (**a**) the working principle; (**b**) the photo; (**c**) the schematic of the test circuit.

The inertial switch can be divided into high-*g* inertial switches and low-*g* inertial switches according to the different load environment applied. On the one hand, the high-*g* inertial switches generally refer to the inertial switches whose threshold acceleration range is from several hundred *g* to tens of thousands *g*. The high-*g* inertial switches are mainly applied in the harsh environment of high load and high impact, such as in the military. A high-*g* switch also needs to have better anti-jamming ability and impact resistance. Non-silicon surface machining technology is often adopted in inertial switches. The structure materials and substrates with high strength are used to prevent fracture failure and disengagement of bond wires. Xu et al. [83] developed a multi-directional MEMS inertial switch with shock-resistance. It can resist ultra-high *g* acceleration (about 100,000 *g*) in the reverse sensitive direction. The schematic diagram is shown in Figure 2. The design of the constraint structures can prevent false trigger caused by the rebound of the proof mass. Moreover, the insulating quartz substrate is beneficial to improve the impact resistance and thermal stability under ultra-high *g* acceleration. The proposed MEMS switch is expected to be installed in devices in Internet of Things systems (IoT) to monitor shock and vibration from the external environment. On the other, low-*g* inertial switches, widely used in the aviation and automotive industries, have acceleration responses ranging from several milli *g* to hundreds of *g*. Based on a feasibility study, Lior et al. [84] proposed an idea of using a pair of bistable beams to suspend the proof mass and to sense the acceleration, in which the switch can be closed under sub-*g* inertias. Nam Lee et al. [85] have developed an inertial switch with a threshold acceleration of no more than 10 *g*, and it can withstand unexpected shocks of up to 1000 *g*, making it suitable for harsh military environments.

**Figure 3:**
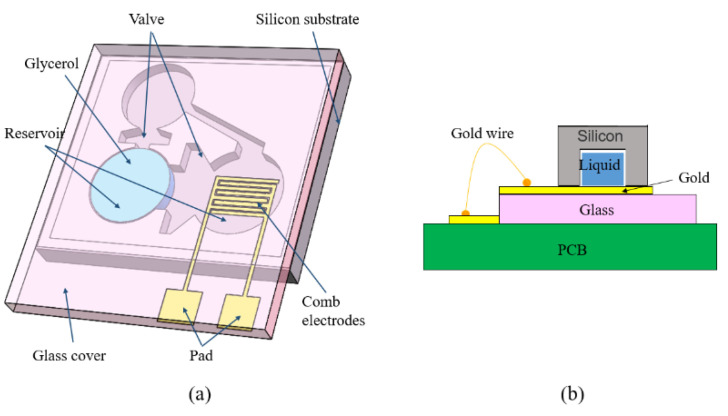
The schematic of a micro-drop inertial switch (Huang et al. 2013 [6]): (**a**) the overview; (**b**) the packing.

Rigid electrodes of MEMS switches have the problems of short contact time and signal bounce. To improve contact stability, many methods have been proposed in terms of structure design and materials selection. Huang et al. [6] proposed a time-delay MEMS switch for safety and arming system. As shown in Figure 3, when the acceleration reaches or exceeds the predicted threshold, the working fluid will flow toward the induction reservoir through the capillary valve. After the delay time, the capacitance between electrodes changes and the switch is turned on. The measurements show that the designed switch can realize a delay time of 4.1~10.9 s. Because of the wedge-shaped channel design, it is difficult for the droplet to flow back from the induction reservoir, so the switch can output a stable switch-on signal. This microfluidic switch has simple preparation technology and high reliability, but the working temperature range of glycerol is narrow (−17.8–290 °C). Liu et al. [86], Yoo et al. [5], Li et al. [7] also designed micro-fluid inertial switches based on the principle of inertial flow. They used mercury or an ultra-low temperature conductive fluid as working fluids. Among them, mercury has excellent electrical conductivity, but it is volatile and only suitable for low-*g* value environments. Liquid metal switches greatly enhance the contact effect, but the choice of working fluids and how to maintain their state stability are tricky issues.

**Figure 4:**
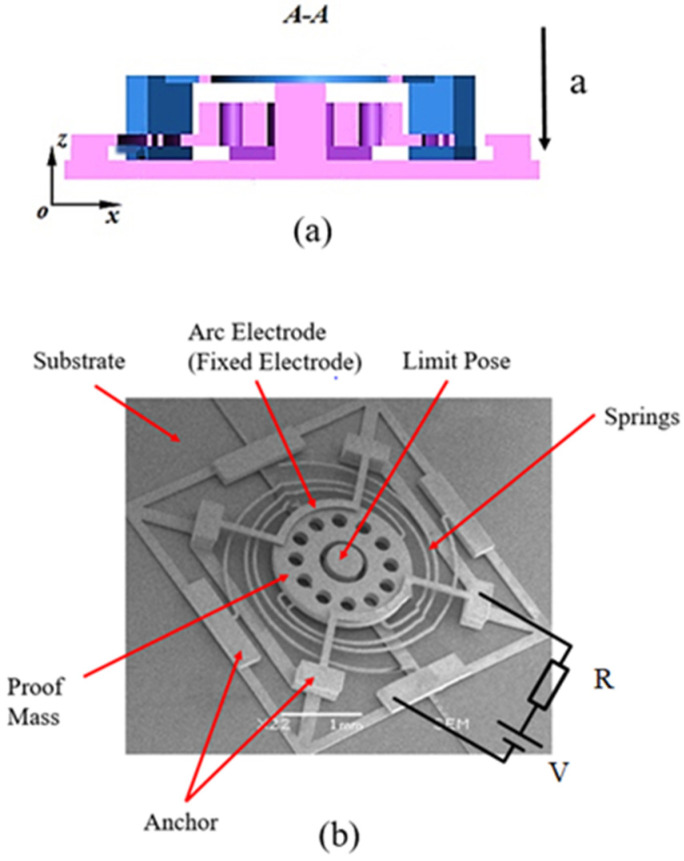
The contact-enhance inertial switch (Du et al. 2020 [8]): (**a**) the cross section of the switch model; (**b**) the SEM image of the switches.

In another way, during the contact process, the deformation of the flexible electrodes (i.e., the fixed electrode or the movable electrode) can provide a buffer for the collision contact between the electrodes, so as to prolong the contact time. Du [8] developed an inertial switch with a low stiffness-fixed electrode for extending the contact duration in 2020 (see Figure 4). The fixed electrode was designed in an arc to reduce its stiffness. The inertial switch was fabricated by UV-LIGA, in which the method of width compensation was adopted to improve the fabrication accuracy. The result showed that the contact time can reach 260 μs when the designed switch is triggered by 32 *g*. At the same time, Xu et al. [87] proposed a vertically driven MEMS inertial switch with a flexible structure. The designed switch can achieve 125 μs contact time at 288 *g* acceleration. By contrast experiment, the conclusion has been proved that the extension of contact time can be achieved by reducing the stiffness of the fixed electrode, especially its thickness.

In addition to the method of utilizing flexible structure to extend the contact time as described above, some literature mentioned carbon on nanotubes (CNTs) as electrode contact materials [9,88,89]. CNTs are suitable for use as contact materials due to their excellent mechanical and electrical properties. Lee et al. [9] have fabricated an inertial switch with CNTs-to-CNTs contact. When the moving electrode collides with the fixed electrode, the elastic deformation of the CNTS greatly increases the contact time. The results showed that under the same conditions, the contact time of the CNTs-based switch was 114 μs, while that of the switch without CNTs was 7.5 μs. Its lifetime is tested to be longer than 57 thousand cycles. The electrothermal actuator and bistable mechanism are used to form the initial gap between electrodes.

Further, there are also some methods taking advantage of the latching mechanism to maintain the switch-on state. The common methods include the mechanical locking mechanism and the bistable mechanism. Mechanical locking switches utilize a pair of mechanical locks to buckle electrodes together [90,91]. The design of the mechanical locking switch requires consideration of an unlocking mechanism to release the movable electrode, without which the switch will remain on after being triggered. The design criteria of bistable bending beams can be found in [92,93]. Zhao et al. [94] developed a bistable inertial switch based on the structure of an inclined buckling beam. Go et al. [95] fabricated a bistable inertial switch using a SiO_2_/p^+^-Si bimorph with residual stress. Frangi et al. [96] also developed a similar bistable structure. These switches require to be applied to the opposite force to restore themselves to the original position. It is worth mentioning that apart from being widely used in inertial switches, the latch mechanism is often used in a variety of other switches to reduce power consumption and enhance switch closure, as described in the following sections.

### 3.2. Electrostatic Switches

The principle of electrostatic actuation widely used in MEMS is the utilization of electrostatic attraction between charged objects to cause the deformation or displacement of objects. Electrostatic switches have been widely studied, and long-term reliability is the main problem that restricts their development. The experiments conducted by G Goldsmith [97] show that the lifetime of the capacitive switch is exponentially related to actuation voltage. For every 5–7 V reduction in the actuation voltage, the switch lifetime is extended by 10 years. Reducing actuation voltage not only extends switch lifetime, but also facilitates its use in wireless devices [98].

**Figure 5:**
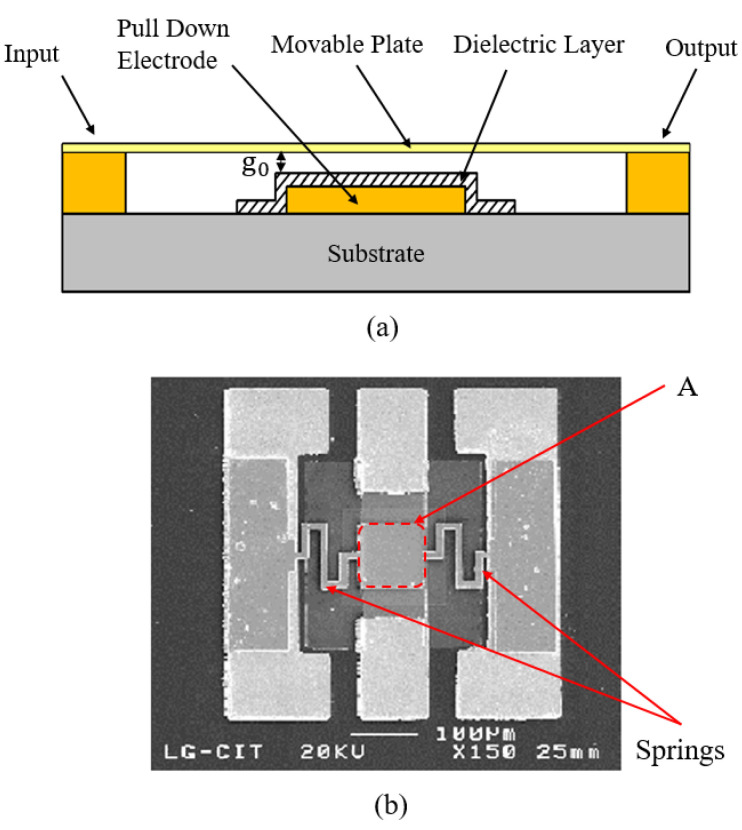
(**a**) The schematic of electrostatic capacitive MEMS switches; (**b**) The SEM image of a radio frequency (RF) MEMS capacitive switch fabricated by J.Y.Park et al. (2001 [10]).

The schematic of a generic switch with electrostatic actuation is shown in Figure 5a. For electrostatically actuated switches, the Coulomb force is proportional to the applied voltage. When the Coulomb force exceeds the elastic restoring force of the movable electrode, it suddenly collapses onto the fixed electrode. This phenomenon is called pull-in instability [99] and the corresponding potential difference, which is a critical value, is called the pull-in voltage. The thin dielectric layer exists to form a coupling capacitor between the electrodes [100]. When the two come into contact, the coupling capacitance becomes so large that the switch is turned off. In order to obtain a large on/off capacitance ratio, the dielectric layer is usually made very thin (not exceeding 300 nm), while capacitive switches typically require 30–80 V. At this high field intensity, charge trapping is prone to occur, which causes the dielectric layer charging [101]. The accumulation of charging charges will eventually prevent the plate from being pulled down or cause the plate to adhere to the dielectric layer, resulting in switch failure. The reliability of the capacitive switch can be improved by optimizing dielectric materials and decrease the driving voltage. Equation (2) gives a widely cited formula for calculating the pull-in voltage of a vertically driven capacitor switch with a traditional rigid plate:

(2) $$V_{p}=\sqrt{\frac{8Kg_{0}{}^{3}}{27\varepsilon_{0}A}}$$

where *K* is the spring constant of the moving structure in the desired direction of motion, $g_{0}$ is the initial gap between electrodes, $\varepsilon_{0}$ is the dielectric constant, and *A* is the area applied to the movable electrode. The equation intuitively shows the influence factors of actuation voltage. Reducing the spring constant *K* has been shown to be the most effective way to reduce the voltage structurally and subsequently prolong the switching time [102]. Figure 5b is a RF MEMS capacitive switch fabricated by J.Y.Park et al. [10]. Strontium titanate oxide (SrTiO_3_) with a high dielectric constant is used as the dielectric layer. In order to reduce the actuation voltage, comparative experiments have been carried out from the aspects of spring geometries, transmission line surface materials and initial gap height. The experimental results showed that the switch with serpentine springs has the lowest actuation voltage (i.e., 8 V) through reducing the spring constant without taking up too much space. This RF switch can be widely used in wireless applications.

It should be noted that unlike the rigid plates mentioned above, the research on elastic plates has been on the rise in recent years. For instance, micro-curved plates can exhibit bistable behavior under appropriate driving force [103]. Although it is difficult to fabricate curved bistable microplates in the MEMS process, it is still a promising research direction. For instance, Asher et al. [104] presented a self-molding forming technique for extruding non-planar thin-walled microstructures with a soft foam stamp. The bistable microcap was fabricated and its bistability was verified for the first time. A similar fabrication method can be found in [105]. Compared with the rigid flat plate, the bistable curved plate used in the switch has the features of reducing power consumption, improving response speed and increasing output displacement. The design method of bistable curved circular plates driven by electrostatic force can be found in [106,107] to determine the initial geometric parameters.

**Figure 6:**
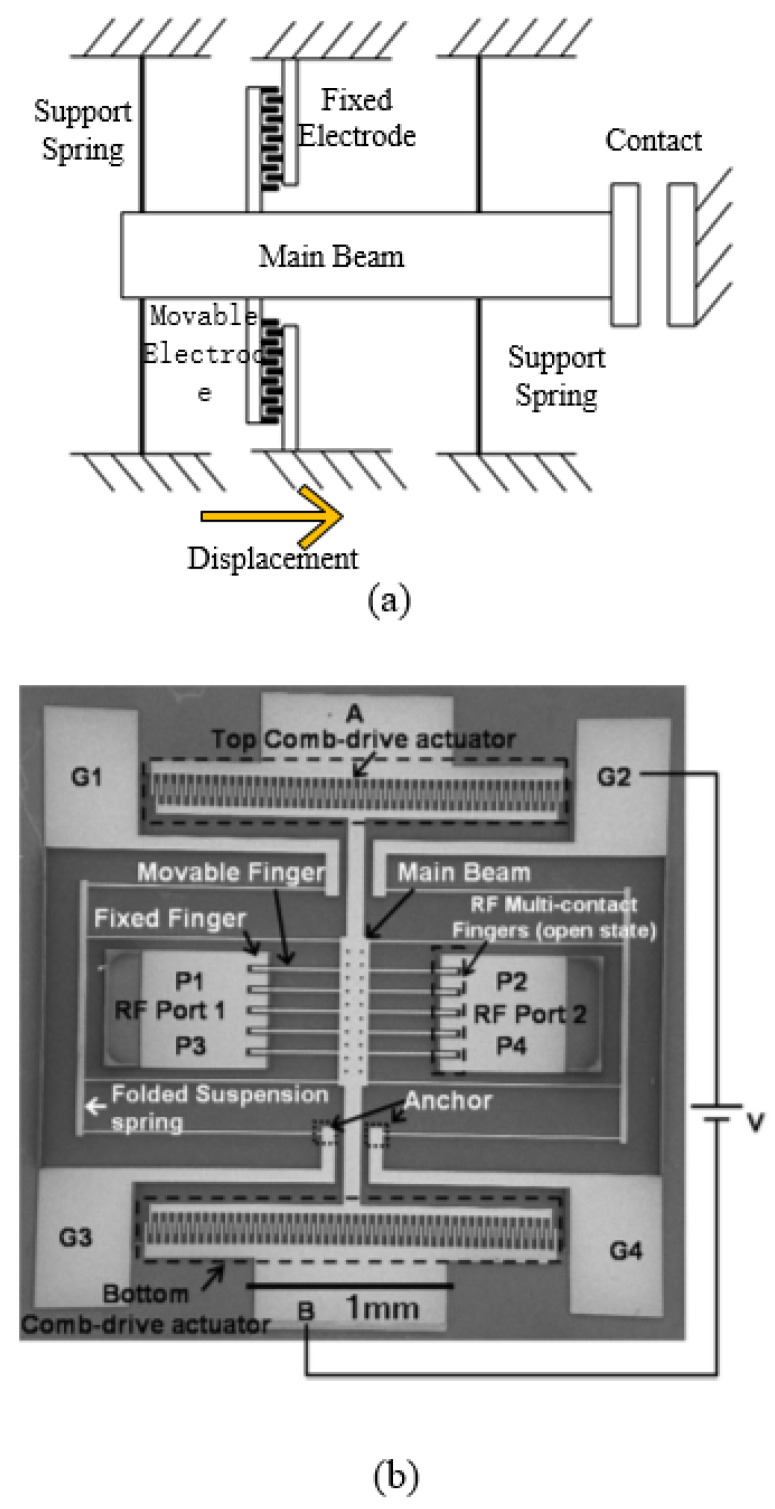
The electrostatic comb-driven multi-contactor MEMES switch (Almeida et al. 2007 [39]): (**a**) the schematic; (**b**) the SEM image.

The structure of a capacitance switch is simple, but the displacement range is limited by the nonlinear behavior of electrostatic force. In order to extend the stable stroke of the electrostatic drive (≥10 μm), the comb-like actuator is usually used. Electrostatic comb-driven switches are laterally actuated [108] and their contact modes are generally designed to be resistive. In some case, the output force is independent of displacement. Almeida et al. [39] fabricated a comb-like electrostatic multi-contactor RF MEMS switch which can be simplified, as shown in Figure 6a. The switch consists of a movable main beam, five movable fingers and six fixed fingers. When a DC voltage is applied to one of the comb-drive actuators, the main beam is moved by electrostatic force, causing movable fingers to come into contact with fixed fingers (see Figure 6b). Au was electroplated on the contact surface to reduce the contact resistance. The overall size of the switch is 3 × 3 mm² and the initial gap between electrodes is 10 μm. However, the comb-like structure of the proposed device makes the actuation voltage increase up to 172–220 V, which not only limits its integration with the IC, but also easily leads to adhesion between electrodes [109]. The lifetime of this switch was tested up to 80,000 cycles.

The optimization of electrostatic MEMS switches is mainly achieved by reducing its actuation voltage [110]. Current methods include shape optimization of comb fingers [111], reduction of the driven gap [112] and reduction of the spring constant [102]. Park et al. [113] proposed a laterally capacitive shunt MEMS switch fabricated on an SOI (silicon-on-insulator) wafer. One thousand comb fingers were used with a gap of 2.1 μm. The air was used as both on and off state capacitive coupling switches instead of dielectric material. The actuation voltage of this switch is 25 V. The second way is the reduction of the spring constant. Kundu [12] reported an RF MEMS switch with low actuation voltage. The actuation voltage was reduced from 20 V to 15 V by introducing the concept of a moving bottom plate and analyzing the performance characteristics of such MEMS switches with two movable plates. Chu [114] proposed a method to realize low voltage of electrostatic switches by utilizing the buckling and bending effects caused by residual stress. The minimum voltage of this switch is 10.2 V. Agrawal et al. [115] presented an electrostatically actuated switch with a hollow beam. By comparing it to the switch with solid beam structure, it was found that the driving voltage of the hollow-beam switch was reduced by four times and the chip area was not increased.

The bistable mechanism can be used to reduce the voltage and power consumption of electrostatic switches. For a traditional electrostatic-driven bistable structure, the switching of two ground states generally requires two driving electrodes to apply two opposite loads on the bistable structure [116,117,118], thus increasing the chip area. Kwon et al. [119] proposed a spatula-shaped comb actuator to realize the bistable state of the bending beam. The direction of the electrostatic force can be changed by changing the relative position of the movable comb and the fixed comb. This design only requires a single driver electrode, but the overall size is not reduced. In recent years, it has been found that dynamic snap-back can be used to release a latched beam with a single electrode [120], which can reduce the chip area. The principle is to apply a gradually increasing voltage to the bistable beam until the voltage is slightly higher than the pull-in voltage, then suddenly remove it. The beam will return from the latched state to its original state. In addition to the lateral drive, Medina et al. [121] later applied this concept to the out-of-plane actuation and presented a snap-through switch actuated by a bistable bow-like beam. Its bistable structure helps to reduce power consumption. The use of the bow-beam actuator reduces the voltage by 45% compared to the common snap-through switch. Furthermore, the research of a capacitive cantilever beam switch driven by three steady-state electrostatic forces has appeared recently [122]. Symmetry breaking should be paid attention to in the design of a bistable structure [93,123,124]. When the ratio of arch height to arch thickness is greater than a certain value, asymmetric transition will occur, which is a hindrance to the realization of latching.

For comb drive electrostatic switches, there is also a signal partition problem that must be paid attention, namely drive signal and switch signal non-interference. Kang et al. [125] introduced a change in the fabrication process of a comb-driven RF MEMS switch. For this device, a 2 μm thick layer of tetraethyl orthosilicate is deposited and then patterned on the silicon structural layer. The signal pads are then made and contactors coated by electroplating 3 μm Au. In this way, an isolation is formed between the electrostatic drive signal and the on–off signal of the switch.

### 3.3. Electromagnetic Switch

The principle of the electromagnetic micro-switch is that the magnetic movable electrode attracted by the electromagnetic coils moves to the substrate, and then the contacts are sucked together and the controlled circuit is switched on [126]. The state conversion of the switch is achieved by entering a bidirectional DC pulse current into the coils.

**Figure 7:**
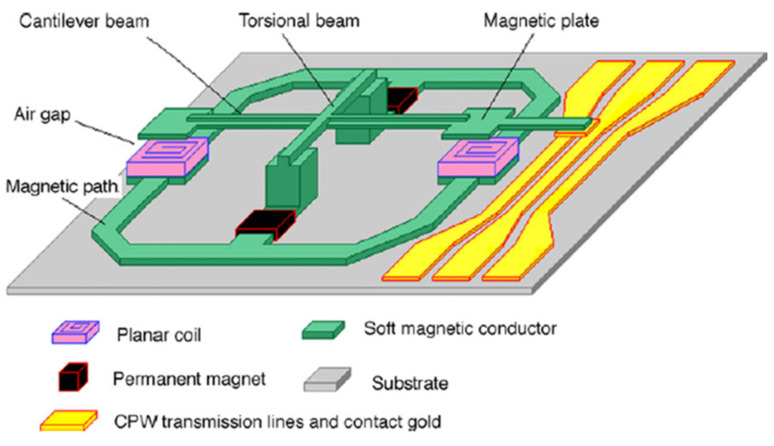
Schematic view of the electromagnetic switch (Zhang 2007 [127]).

In 2007, Zhang [127] reported a high-speed bistable electromagnetic actuator for resistive RF MEMS switches by UV-LIGA technology. A schematic drawing of the electromagnetic actuator is shown in Figure 7. There are many details that can be enhanced in order to improve performance. The application of a torsion beam can improve the restoring force and reliability of the cantilever beam. Quartz glass was chosen as the substrate material here to reduce the substrate power loss. The cantilever beam with a T-shaped cross-section was adopted for larger displacement and reduced mass of the device. The multilevel Cu coils were designed in plane structures to adapt to the MEMS process. ${\mathrm{Al}}_{3}O_{2}$ was sputtered as the insulating layer. The permanent magnets made by precision-machining technology are manually installed into the device. The test results showed that the switch speed is 20 μs at 50 mA pulse current. Under the action of the torsion beam and permanent magnet, a bistable state can be easily realized, thus reducing power consumption. The overall size is 2 mm × 2 mm.

**Figure 8:**
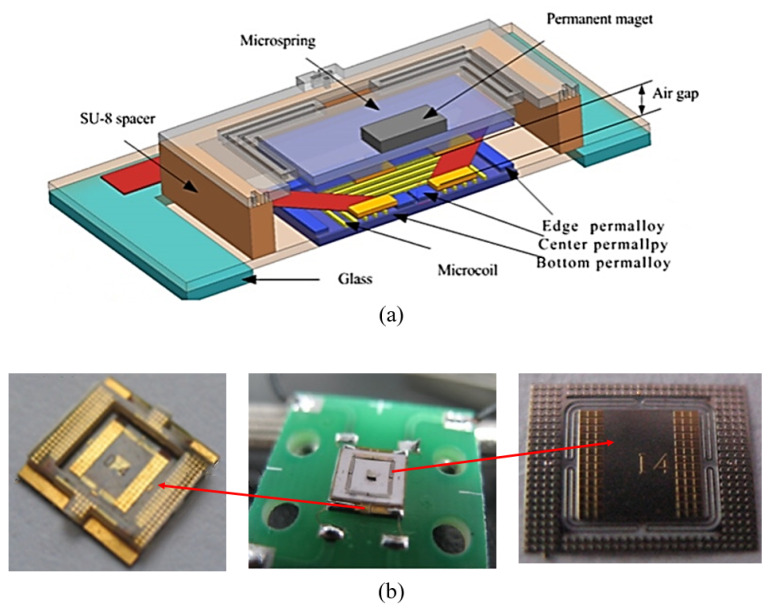
The bistable electromagnetic switch proposed by Miao et al. (2011 [128]): (**a**) the schematic; (**b**) the pictures of the prototype.

Miao et al. [128] proposed an electromagnetic bistable switch by surface micromaching technology on a glass substrate, as shown in Figure 8. In their ingenious design, the switch mainly consists of a coil component and a spring supporting a permanent magnet. The switching of the two steady states is realized by changing the direction of the pulse current in the coil. The top and bottom contactors are made of electroplated Au. By adjusting the width and thickness of the cantilever beams, the spring elasticity can be increased in a limited space, thus reducing the actuation voltage. Polyimide is used to prepare insulating layers. At 5 V pulse voltage, the switch can achieve a response time of no more than 5 ms and an output displacement of up to 380 μm. Unfortunately, this form of microassembly increases the difficulty of operation, and the complex process of the electromagnetic MEMS switch limits its mass production. The chip area of the switch is 6 mm × 6 mm.

$Al_{2}O_{3}$ and polyimide are often used as insulators in electromagnetic MEMS switches. The structure of microcoils has a direct influence on performance of the switch [89], for example, multi-layer coils and the addition of magnetic cores can significantly improve the driving force. A permanent magnet allows the electrodes to remain in contact after the current is removed without additional power apply, through which the switch can achieve a bistable state to reduce power consumption. The actuation voltage of electromagnetic switches is as low as the requirement of the integrated circuit (<10 V), and the switch has fast response speed (<5 ms). It can adapt to a bad environment [128], especially suitable for large displacement (50–400 μm) [70]. The deficiencies of these switches are complex process (some require microassembly) and need to occupy a large chip area (>2000 × 2000 μm^2^). Electromagnetic switches have not advanced by leaps and bounds in the last decade. To greatly improve the applicability of electromagnetic switches, future optimizations should still focus on reducing the power consumption, reducing the size without reducing the electromagnetic force and improving the maturity of the processing technology.

### 3.4. Piezoelectric Switch

As for piezoelectric switches, switching between on and off is achieved by the converse piezoelectric effect of piezoelectric materials [129]. Piezoelectric strains can be either positive or negative, therefore this kind of switch can be used actively to turn the switch off as well as on. Widely used piezoelectric materials include aluminum nitride (AlN), lead zirconate titanate (PZT) and so on.

**Figure 9:**
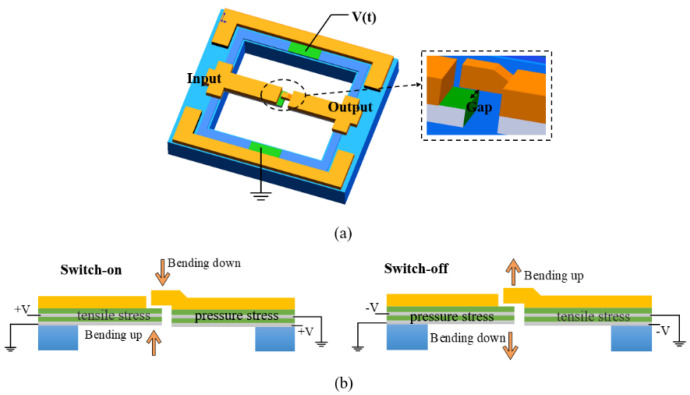
The piezoelectric AlN RF MEMS switches of dual-beam actuation proposed by R. Mahameed et al. (2008 [11]): (**a**) the model; (**b**) the working principle.

R. Mahameed et al. [11] firstly proposed a laminated double-beam RF MEMS switch based on AlN (see Figure 9). Compared with PZT, the fabrication process of AlN is compatible with a CMOS (complementary metal-oxide-semiconductor transistor). The dual-beam design is adopted to essentially compensate for the residual stress in the deposited films, where the bottom layer of AlN is used for driving. The switch can be used actively to turn the switch off as well as on by reversing the polarity of driven voltage. The drive pads are utilized to drive both beams at the same time. In this way, the drive voltage is reduced to no more than 20 V, the contact force doubled, and the switching time reduced by half.

**Figure 10:**
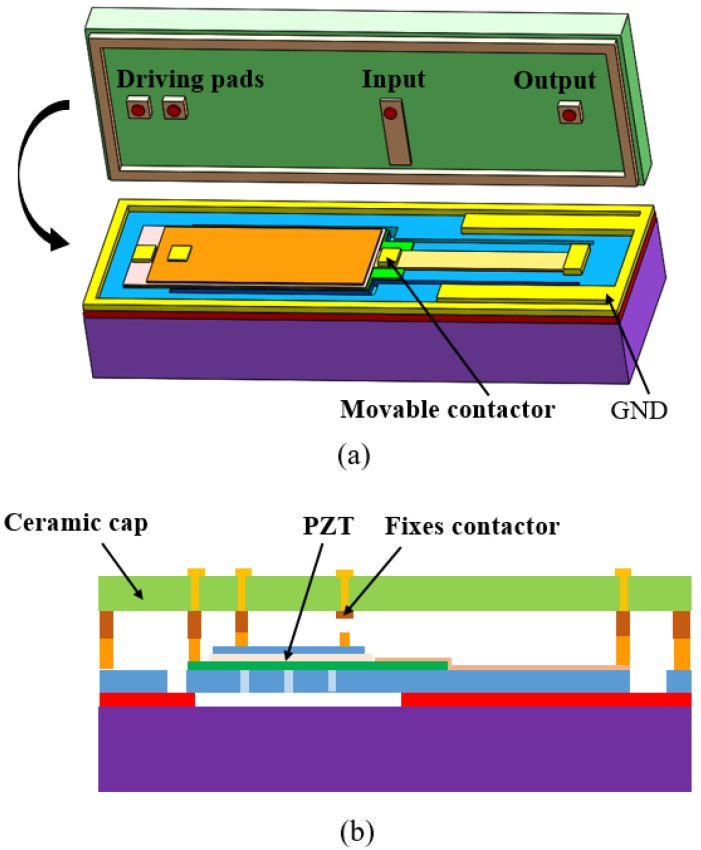
A compact piezoelectric-actuated MEMS switch (Nakatani et al. 2013 [130]): (**a**) the schematic view; (**b**) the photo of the fabricated switch.

Nakatani et al. [130] developed a compact resistive piezoelectric actuated switch, as shown in Figure 10. The silicon beam of the switch was fabricated by opening a window on the device layer of the SOI wafer and then etching the buried silicon dioxide layer with hydrofluoric acid. The PZT film actuator and movable electrode were in turn prepared on the device layer. The ceramic cover plate with a fixed electrode was finally bonded to the SOI wafer to form the initial gap and tightly package the switch at the same time. In the initial state, the uniform 0.5 μm gap between the electrodes makes the structure compact. The fabricated switch can produce a contact force of 1 mN at a driving voltage of 20 V. In this way, the high restoring forces prevent adhesion, and this sealing solution prevents also the organic contamination of the contact surface. These design details help to make the service life of the switch up to 1 billion.

The well-known bistable mechanism is also used in piezoelectric actuation. Manuel Dorfmeister et al. [131] proposed a piezoelectric actuator with a bistable membrane. An SOI wafer with a 2 μm thick device layer was used as the substrate and the AlN layer was deposited as the piezoelectric layer. ${\mathrm{Si}}_{3}N_{4}$ was sandwiched between them as the insulating layer. When the internal stress exceeds the critical value, the film will deflect and remain in its stable state. The film can be converted between two ground states by applying impulse currents in reverse. The AlN bistable piezoelectric film produced a displacement of 10 μm. The bistable membrane design is expected to be applied to piezoelectric MEMS switches to reduce power consumption.

Piezoelectric drive has the advantages of fast response speed (<300 μs) [11] and stable output, even for small displacements (0.5–2 μm). Piezoelectric actuation is the most suitable technique for the biomedical devices of all actuation types [132]. PZT has a high value of the piezoelectric coefficient d_33_ > 100 pC/N, while this of AIN is d_33_ = 5 pC/N, so PZT is most commonly used in the research of piezoelectric switches. In order to reduce the power consumption of piezoelectric switches, bistable mechanisms such as bistable curved microplates can be adopted [133]. The fabrication of piezoelectric materials by the MEMS process is a concern.

### 3.5. Electrothermal Switch

The common thermal-actuated structures are the bimorph structure and bending beam structure (including V-shaped and U-shaped beams). The principle of the bimorph actuator is similar to that of piezoelectric actuators [134,135]. Electrothermal switches based on bending beam actuators, especially V-shaped actuators, have been studied extensively.

**Figure 11:**
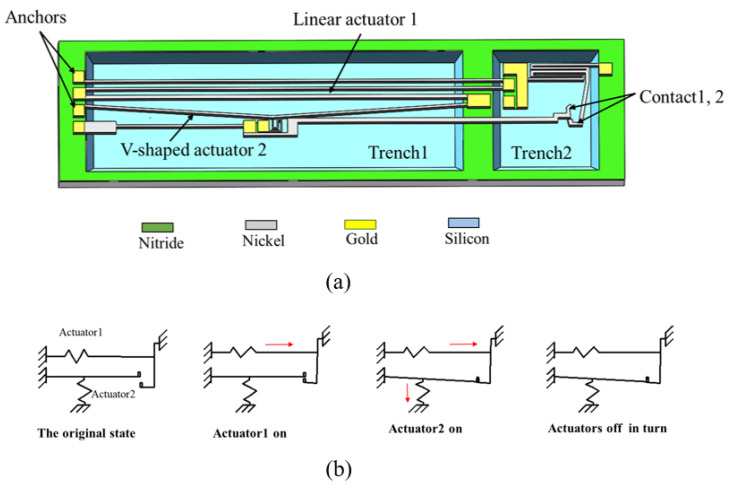
A compact thermal driven MEMS bistable switch (Dellaert D et al. 2015 [40]): (**a**) the schematic; (**b**) the working process.

Dellaert D et al. [40] proposed a compact thermal driven latched MEMS switch, as shown in Figure 11. The main structure of the switch is composed of a linear actuator, a V-shaped actuator, a pair of vertically placed contacts and two levers. The two actuators drive the contactors respectively, and the contactors realize a bistable state by means of mechanical self-locking. The levers are used to slightly amplify the displacement. The combination of the two actuators makes the switch produce a high execution force of 1.33 mN at a small displacement. The use of MetalMUMPs (metal multi-user MEMS processes) technology allows the patterned $Si_{3}N_{4}$ layer to be suspended on the etched trench and to support the 20-μm nickel structure layer. In addition to mechanical support, the silicon nitride layer acts as an insulator to separate the drive current from the switch signal. Au is sputtering on the sidewall of the contactors to lower the contact resistance, which is 0.6 Ω at 10 mA. The prominent features of the switch are to save the chip area through reasonable placement and to reduce the contact resistance through using a metal structure, sputtering Au on the sidewall of the contacts as well as high contact force of 1.33 mN at the switch area of 2020 × 330 μm².

**Figure 12:**
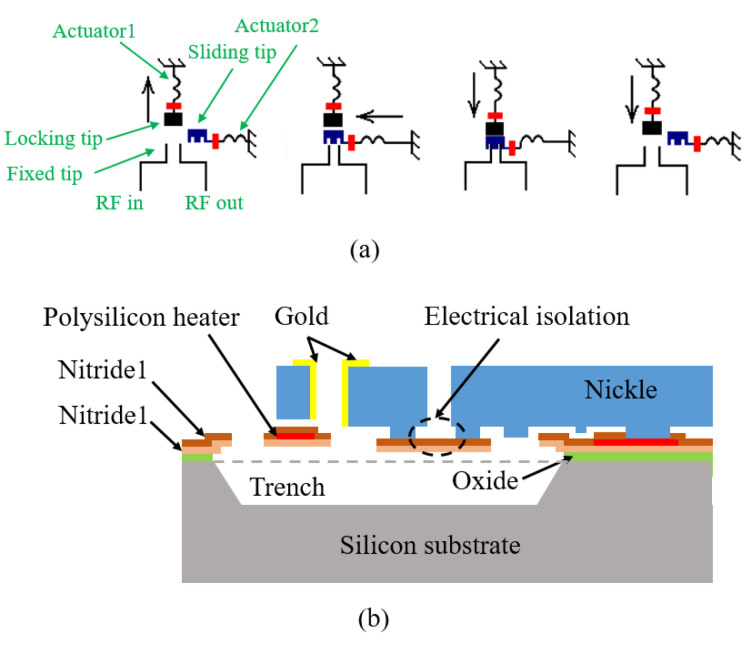
A latching MEMS electrothermal switch (BakriKassem M 2015 [41,42]): (**a**) the working principle; (**b**) the diagram of processing.

Bakrikassem, M. et al. [41,42] proposed an electrothermal mechanical latching switch. Its working principle is shown in Figure 12a. The switch closure and latch are realized through the sequential actions of two sets of electrothermal actuators. The switch is fabricated using the MetalMUMPS process, as shown in Figure 12b. The polysilicon layer acts as a heating resistor. When a current is applied to the polysilicon resistor through the DC pads, the heat generated is transferred to the V-shaped metal actuator structure through the ${\mathrm{Si}}_{3}N_{4}$ layer that wraps it. When the groove on the sliding tip moves to the top of the fixed tips, the latch tip presses the sliding tip down by restoring force and latches the position. The sliding tip acts as a bridge connecting the two fixed tips and closing the circuit. On the one hand, ${\mathrm{Si}}_{3}N_{4}$ protects polysilicon from corrosion by KOH solution used to etch the trench; on the other hand, it separates the drive signal from the switch signal to avoid signal coupling (between the locking tip and the actuator, and between the sliding end and the actuator). The reaction force on the sliding tip produces vertical strain inside the locking tip, which affects the service life of the ${\mathrm{Si}}_{s}N_{4}$ layer below the locking layer. The actuator is fabricated by 20 μm thick Ni connected with an Au-plated tip to enhance the contact effect. The DC power consumption is 250 mW for a displacement of 32 μm in 14 ms.

**Figure 13:**
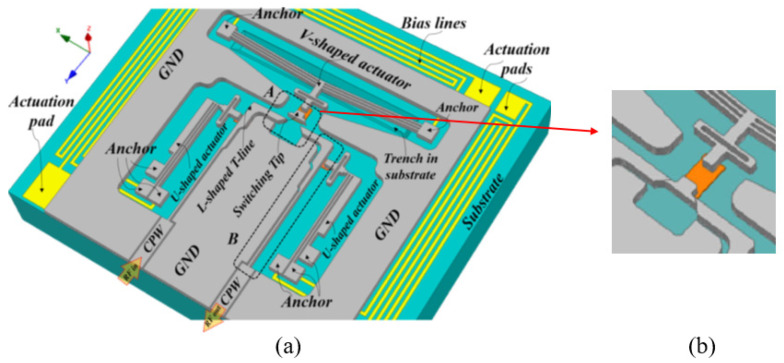
The electrothermal actuated bistable MEMS switch (Zolfaghari P et al. 2018 [13]): (**a**) the schematic view; (**b**) the close-up of the switching tip.

Zolfaghari P et al. [13] have studied a kind of electrothermally driven bistable RF MEMS switch with low power consumption, as shown in Figure 13. The MetalMUMPs technology was also adopted and the structure layer was made of Ni. The switch structure has a symmetrical distribution, with U-shaped actuators linked with movable contacts on either hand and a V-shaped actuator with a movable contactor on the top. The $Si_{3}N_{4}$ layer not only connects the contactors to the U-shaped actuators or the V-shaped actuator, but also insulates the drive signal from the switch signal. U-shaped actuators are used to pull apart the two lower contactors, and V-shaped actuators are used to push the upper contactor forward. Pulse voltages are applied sequentially to the U-shaped and V-shaped actuators, and the switch can be on or off. When the sidewall of the three wedge-shaped contacts fit together, the mechanical forces they generate latch the position of the switch. Au film of 1–3 μm is deposited on the surface of the contactors by electroplating to reduce contact resistance. The driving voltages are no more than 1 V, and the contact resistance is 0.028 Ω.

**Figure 14:**
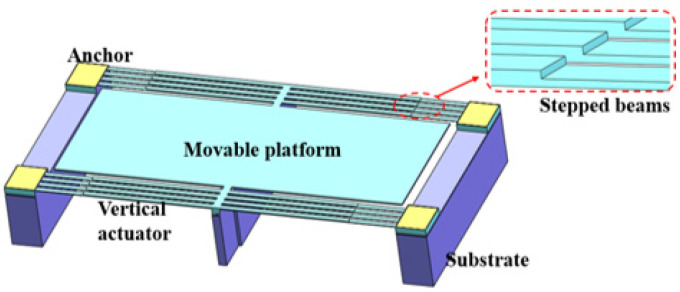
An electrothermal actuator with stepped beams (Kim 2013 [136]).

There are also some structural variants of actuators for electrothermal switches. For instance, Kim et al. [136] proposed an actuator with stepped beams fabricated on an SOI wafer in 2013 (see Figure 14). When current is applied from the anchors of the beam array, the difference in section thickness of the beams causes a bending motion in the vertical direction. This actuator can be used for vertically actuated switches.

The thermal actuation overcomes the weakness of a great dependence on the gap compared with electrostatic and electromagnetic actuation, and has large output displacement (laterally actuated >30 μm) [137] and output force (>500 μN) [138]. The main disadvantages of thermal switches are slow response (a few thousand μs) and high power consumption (a few hundred mW). A great deal of effort has been made to address the shortcomings of electrothermal switches. Similar to switches with other driving principles, bistable systems are frequently used for their low power consumption [13,40,41,42,139,140]. The response speed of MEMS switches can be improved by the actuator optimization of geometry, material properties and driven voltage. Electrothermal switches are basically resistive switches. Thus, low contact resistance is the key performance of this type of switches. In the switch fabricated by MetalMUMPS process, 2–3 μm Au layers can be electroplated by sputtering the seed layer first. Alternatively, in the case of SOI wafers, the non-perpendicularity of sputtering can be used to sputter the Au directly on the contact surface of the structural layer. Oh C [141] mentioned that: firstly, the sample is fixed and the contact surface on one side is sputtered Au, and then the side wall of the other contact surface is coated by rotating the sample 180° along the normal direction of the substrate. In this way, the metal layer sputtering of the two contact surfaces can be better realized. Signal interference occurs if the drive signal and the switch signal are not isolated. Generally, an electrical isolation layer is sandwiched between two layers of the driving structure and contact. The electric isolation layer is usually the ${\mathrm{Si}}_{3}N_{4}$ layer [40,41], or the ${\mathrm{SiO}}_{2}$ layer which is formed by plasma-enhanced chemical vapor deposition (PECVD) [142] and then patterned.

### 3.6. Multiple-Actuation Switch

All of the above drive switches have their advantages and disadvantages. For example, the electrostatic drive consumes extremely low power and has a simple fabrication process, but the electrostatic actuation requires 20–100 V or even higher driving voltage, which is not conducive to system integration and long-term reliability. Thermal actuation and electromagnetic actuation can generate greater force and output displacement than electrostatic actuation, and the required voltage generally does not exceed 10 V. However, continuous power supply results in large power consumption. In this case, various improvements have been discussed, including reducing the voltage of the electrostatic drive, increasing the switching speed and the displacement of the capacitor switch by employing a bistable film, and reducing the power consumption of the electrothermal and electromagnetic actuation by latching mechanisms. In addition to these, there are some efforts to combine multiple drivers and leverage their strengths while avoiding their weaknesses.

Cho [143] reported a MEMS switch that combines electrostatic and electromagnetic actuations, which means using electromagnetic force for switching and electrostatic force for state retention. Driven by the electromagnetic force, the displacement is linearly related to the applied current. The initial gap of the two contacts is 10 μm, the driving voltage is 2 V and the holding voltage is 3.7 V. The transient response indicates that the switching time from off to on is less than 110 μs, while the switching time from on to off is about less than 380 μs. The power consumption is only 40.3 μJ. Chae et al. [144] proposed an RF MEMS switch that takes advantages of electrostatic and electrothermal drives. The driving voltage is 0.3 V and the holding voltage is 15.4 V. The switching time from off state to on state is 47 μs, while the switching time from on to off is 4.5 μs. The power consumption is 3.24 μJ. Similar work can be found in [144,145,146,147,148]. The actuation voltage and power consumption of these multi-drive switches are both decreased. The downside, of course, is the increased chip area and complexity.

### 3.7. MOEMS Optical Switch

The MOEMS optical switch is not a kind of conventional MEMS mechanical switch, so this review only mentions it in passing. It is actually a technology of optical path switching employing MEMS components such as movable micromirrors and movable resonators [149,150]. Typical MOEMS optical switches consist of input/output optical fibers, movable micromirrors and actuators. By apply the appropriate driving voltage, the micromirror array can move along a straight line or twist at an angle so as to realize on and off of the optical path. The driving principle of MOEMS optical switches can be electrostatic, electro-thermal or other actuations as mentioned above. For instance, Sun et al. [151] proposed a 2 × 2 electrostatic-driven optical switch with a torsion beam. The upper and lower electrodes form a flat capacitive structure with an electrical isolation layer between the two and a micromirror on the upper electrode. When no voltage is applied, light beams are reflected by the micromirror. When the driving voltage is applied, the upper plate bends, thus changing light beams from a reflected state to a transmitted state. The optical switch is processed by MEMS switch technology, which has the advantages of miniaturization, mass quantization and compatibility with large-scale integrated circuits. It plays an important role in optical fiber communication.

## 4. The Development Trend of MEMS Switches

For a more intuitive view of the development direction of MEMS switches over the past 20 years, the switches and their characteristics in some representative articles are listed in the following Table 3 in chronological order.

As can be seen from Table 3, passive inertia switches remain a research hotspot benefiting from their particular application market. As for active switches, electrostatic and electrothermal switches have developed more rapidly in recent years. In the choice of contact mode, the resistive switch is the mainstream relative to the capacitive switch. For resistive switches, Au-Au contact is mainly selected from the point of view of reducing contact resistance. Further from the point of view of reliability, there are but few switches using other contact materials. The performance optimization of various switches mainly focuses on the following aspects: (1) Reduced power consumption. Of all, electromagnetic and electrothermal actuation require relatively high power consumption. At present, the main method to reduce power consumption is to use a bistable mechanism due to their ultra-low power consumption, as both ground states remain stable without any energy supply [13,93,95,122]. (2) Enhanced contact performance. For resistive switches, switches need to have good contact effect to accurately identify switch signals. The extension of contact duration can be achieved by decreasing the stiffness of electrodes; utilizing latching mechanisms including mechanical self-locking, a bistable mechanism and a wedge-shaped channel, etc. [4,5,6,7,8,74,77,80]. The contact resistance can be reduced by using the contact materials with low resistivity [13,57] and increasing the contact force [80,165]. (3) Process technology. The current fabrication technologies of MEMS switches are mainly divided into SOI bulk micromachining and metal surface micromachining. The excellent mechanical and electrical properties of metals facilitate the transfer of switch signals, which leads to the wider application of MetalMUMPs technology in the fabrication of MEMS switches [8]. However, silicon-based MEMS switches are also being studied persistently due to their unsurmountable merits, that is, high shape precision and simple and mature processes, which are more suitable for mass production. (4) Impact resistance. This specification, especially for inertial switches, can be met by optimizing the structure and substrate materials [59]. (5) Miniaturization. One of the reasons passive switches stay hot is their smaller size when compared to active inertial switches. The thermal actuator can achieve a large displacement output, but its slender shape takes up a large chip area. Therefore, novel designs tend to be developed to make the structure more compact [80]. (6) Improved durability. The long-term lifetime of MEMS switches has been improved in many ways, such as by adopting dielectric-free switches to avoid dielectric charging, choosing gold alloys or contact materials combining gold with other materials to reduce contact degradation, enhancement of heat dissipation of devices, reducing residual stress in structural design and process technology, and strengthening seals to prevent moisture and pollution.

Through the above analysis, it can be found that MEMS switches are distinguished by miniaturization, low power consumption and intelligence, but there are still some problems that need to be urgently solved. Typical problems include: (1) Complex processing technology. There are manifold MEMS processing steps, and some still need to be manually completed, where subjective error is big and quality consistency is difficult to guarantee. (2) Lower reliability [166]. Reliability is a key performance in switch alignment applications. Due to the repeated use of switches and environmental factors, MEMS switches are prone to failure. Although MEMS switches are increasingly needed in civil and military applications, their reliability is often neglected in design. To date, the related failure modes and mechanisms have not been fully explored. In addition, there is also a lack of unified standards to check the reliability of switches, such as lifetime, mechanical properties and so on. (3) High packaging cost. The packing of the switch has always been a conundrum [167,168]. The packaging cost of switches is often much higher than the manufacturing cost, which restricts their mass quantification.

## 5. Summary and Outlook

Over the past decades, various MEMS switches have been designed and fabricated. In this paper, the structural characteristics, relative strengths and weaknesses of switches based on different actuation principles are reviewed. We have also discussed the efforts of various groups to improve the performance of MEMS switches using different techniques. Inertial switches have a significant number of application requirements, and their future development is mainly in the direction of multi-axis sensitivity, high reliability, high impact and load resistance. In terms of active switches, electrostatic and electrothermal switches are more widely used at present on account of their excellent performance. For better use, the optimization of these two type of switches will remain an interesting topic. Furthermore, with the discovery of new materials and the maturity of technology, it is believed that piezoelectric switches will win a place in the field of MEMS switches. Each switch has its advantages and disadvantages, so the choice of driving principle and structure of the switch should be combined with its application requirements. As researchers gradually shift their attention concerned with performance optimization to the improvement of reliability, MEMS switches will gain significant development.

## Figures and Tables

**Table 1:** Failure mode analysis of MEMS switches.

Failure Mode	Failure Factors	Improvement Methods
Creep	Temperature, power, interior stress	Creep resistant alloy; improving heat dissipation;
Stiction	Humidity, adhesion force, power	Reducing contact area; choosing harder contact materials; reducing the power
Dielectric charging	Electric field intensity, temperature, humidity	Lower actuation voltage; signal isolation; changing the dielectric
Fracture	Repeated loading, shock	Reducing stress; change the composition of alloys; shock absorption
Wear	Repeated contact	Increasing the hardness of the contact material
Layered	Temperature change, residual stress, microparticles	Improving temperature stability; transition layer to increase adhesion
Failure of package	Temperature change, impact	Shock absorption; heat dissipation

**Table 2:** Performance comparison of MEMS switches with different driven actuation.

Mechanism	Inertial	Electro-Static	Electro-Magnetic	Piezo-Electric	Electro-Thermal
Size (μm)	~$3000^{2}$	$100^{2}-2000^{2}$	$2000^{2}-6000^{2}$	$200^{2}-2000^{2}$	$300^{2}-2000^{2}$
Fabrication process	Simple	Simple	Complex	Complex	Medium
Actuation voltage (V)	/	20–200	<10	3–20	<15
Power consumption (mW)	NZ ^1^	NZ	100–200	NZ	60–250
Switch speed (μs)	300–1000	<200	20–1000	10–300	300–10,000
The output force (μN)	Uncertain ^2^	50–1000	50–200	50–800	500–4000
Durability	>$10^{6}$	$10^{8}-10^{9}$	~$10^{8}$	~$10^{8}$	$10^{6}$

^1^ NZ: near zero; ^2^ Uncertain: related to structure and acceleration.

**Table 3:** Trait comparison of MEMS switches in different periods.

Authors [Ref] (year)	Driving Principle	Contact Form	Processing Technology	Stroke (μm)	Contact Force (μN)/Acceleration	Contact Resistance (Ω)	Size (μm²)	Driving Voltage (V)	Power Consumption (μJ/mW)	Switching Time (OFF–ON) (μs)	Reliability (Cycles)	Application
Park, J. [10] (2001)	Electrostatic	Capacitive	Metal surface micro-machining	_	/	/	>400 × 400	8	0	/	/	RF MEMS
Robert, P. [147] (2003)	Thermal + electrostatic	Contact	_	_	_	_	400 × 50	10	8	200	>$10^{9}$	RF MEMS
Wang, Y. [152] 2004	Electrothermal	Contact	Silicon surface-micromachining	_	725.7	_	300 × 100	3	60–100	300	$10^{6}$	Microrelay
Almeida, L. [39] (2007)	Electrostatic	Contact	Metal-MUMPs	10	_	0.95–1.9	3000 × 3000	172–220	_	_	$8\times 10^{5}$	RF MEMS
Zhang, Y.H. [127] (2007)	Electromagnetic	Contact	Non-silicon surface micro-machining	17	_	_	2000 × 2200		_	20	_	_
Mahameed [11] (2008)	Piezoelectric	Contact	Non-silicon surface micro-machining	_	0–32	5.4	200 × 200	5–20	_	1–2	_	RF MEMS
Park, J. [129] 2009	Electrostatic	Non-contact	SOI bulk micromachining	_	25	_	4000 × 5000	25.00	_	300	$10^{9}$	RF MEMS
Patel, C.D. [153] (2010)	Electrostatic	Contact (Au-Ru)	Silicon surface-micromachining	0.85	800–1800	1.5	155 × 130	75–90	_	6	_	RF MEMS
Cho [143] (2010)	Electromagnetic + electrostatic	Contact	Non-silicon surface micro-machining	_	46.2	0.42	400 × 250	<4.3	15.4 μJ	447	1.66$\times 10^{8}$	RF MEMS
Miao [128] (2011)	Electromagnetic	Contact	Non-silicon surface micro-machining	380	_	_	6000 × 6000	5	25 mJ	4960	_	Communication facilities
Patel, C.D. [154] (2012)	Electrostatic	Contact	All-metal surface micromachining	0.55	1200–1500	1–2	250 × 250	100	_	5.5	>$10^{8}$	RF MEMS
Lee [9] 2012	Inertial	Contact	SOI bulk micromachining	16	33 *g*	~185	_	/	_	_	>5.7 $\times 10^{5}$	Commercial applications e.g., geriatric health care system
Song [155] (2012)	Electrostatic	Contact	Silicon surface-micromachining	12.5	_	0.005	_	40	_	230	4.9 $\times 10^{5}$	Power-switching application
Czaplewski [156] (2012)	Electrostatic	Contact	Non-silicon surface micro-machining	_	3.5		140 × 150	80	_	_	$10^{8}$	_
Huang [6] (2013)	Inertial	Capacitive	Silicon bulk micromachining	_	44–263 *g*	/	_	/	_	_	_	Safety and arming system
Czaplewski [157] 2013	Electrostatic	Contact (RuO2–Au)	Non-silicon surface micro-machining	3	35	<4	228 × 85	120	_	_	$10^{9}$	RF MEMS
Zhanwen, X. [4] (2014)	Inertial	Contact	Non-silicon surface micro-machining	8–40	350 *g*–500 *g*	_	2800 × 2800	/	_	_	_	Military weapons and industrial applications
Gerson, Y. [74] (2014)	Inertial	Contact	Silicon bulk micromachining		~300 *g*	1.7–2.6	2600 × 2600	/	_	<120	_	-
Nakatani, T. [130] (2014)	Piezoelectric	Contact	SOI bulk micromachining	0.5	1000		1600 × 1100	20	_	_	$10^{8}$	-
Xu, Y. [15] (2015)	Electrostatic	Capacitive	Silicon surface-micromachining	1	/	/	320 × 120	14	_	_	_	Frequency reconfigurable antenna application
BakriKassem, M. [42] (2015)	Electrothermal	Contact	Metal-MUMPs	32	3200	2.4	2000 × 1100	12	250 mW	14,000	_	Latching RF MEMS
Pirmoradi, E. [140] (2015)	Electrothermal	Contact	Silicon surface-micromachining	>5	_	_	_	6	98.78 mW	700	_	RF tuning and switching applications
Zhou [158] (2015)	Inertial	Contact	Metal surface micro-machining	110	_	_	_	_	_	_	_	_
Zhang, Q. [75] 2016	Inertial	Contact	Non-silicon surface micro-machining	20	150–350 *g*	2.35	_	/	_	300–600	_	Remote detection of vibration shock
Angira, M. [159] (2016)	Electrostatic	Capacitive	Silicon surface-micromachining	2	/	/	310 × 90	6	_	_	_	RF MEMS
Lee, Y. [90] (2016)	Inertial	Contact	Silicon surface-micromachining	9	43.7 *g*	/	1800 × 3200	/	_	_	_	Airbags, parachutes, military devices, etc.
Khadeijeh K [109] (2016)	Electrostatic	Contact	Silicon surface-micromachining	0.8	20,000	0.0018	120 × 60	6.27	_	_	_	RF MEMS
Dellaert, D. [139] (2016)	Electrothermal	Contact	Metal-MUMPs	41–71	_	_	2000 × 2000	20–24	_	_	_	Automated distribution frame
Xu [83] (2017)	Inertial	Contact	Non-silicon surface micro-machining	_	>10,000 *g*	9.09	_	/	_	<1000	_	Shock vibration monitoring sensor for IoT
Tomoaki Kageyama [27] (2017)	Electrostatic	Contact (Au-Au/CNTs)	Silicon surface-micromachining	_	_	_	_	90	_	_	9100	RF MEMS
Joshitha, C. [28] (2017)	Electrothermal	Contact	SOI bulk micromachining	20.7	157	_	200 × 200	14	_	_	_	_
Kashani Ilkhechi, A. [37] (2017)	Electrostatic	Contact	Metal-MUMPs	3.7	107	_	_	50	_	79	_	Antenna switch applications
Liu [160] (2017)	Electrostatic	Contact	Silicon surface-micromachining	_	/	5	400 × 300		_	30.4	$10^{8}$	RF MEMS
Lee, H.N. [85] (2017)	Inertial	Contact	Non-silicon surface micro-machining	6.21	10 *g*	_	_	_	_	_	_	Military applications
Zolfaghari [13] (2018)	Electrothermal	Contact	Metal-MUMPs	12–32	950	0.028	_	0.5–0.9	0.56mW	500	_	RF MEMS
Du [22] (2018)	Inertial	Contact	Non-silicon surface micro-machining	>38	40 *g*	_	3870 × 3870	_	_	_	_	Automotive airbags
Pustan, M. [45] (2018)	Electrothermal	Contact	Silicon surface-micromachining	0.6	_	_	1200 × 1000	_	_	490	_	_
Shekhar, S. [161] (2018)	Electrostatic	Capacitive	Non-silicon surface micro-machining	_	/	/	_	4.8	<1 mW	33	$10^{7}$	5G applications
Xi [21] (2019)	Inertial	Contact	Non-silicon surface micro-machining	_	400 *g*–700 *g*	_	_	/	_	_	_	Direction detection
Desireh [32] (2019)	Electrothermal	Contact	Metal-MUMPs	2.6	_	_	_	11	_	_	_	Power Limiter Applications
Ansari [36] (2019)	Electrostatic	Capacitive	Silicon surface-micromachining	/	/	/	_	2.4	_	~10	_	Communication facilities
Du [8] (2020)	Inertial	Contact	Metal surface micro-machining	50	26 *g*	_	3850 × 3850	/	_	<2240	_	Airbag restraint system
Chea [144] (2020)	Electrothermal + electrostatic	Contact	Silicon surface-micromachining	4.7	_	1	300 × 160	15.4	3.24 μJ	47	2.1$\times 10^{7}$	RF MEMS
Krakover, N. [162] (2020)	Inertial	Contact	SOI bulk micromachining	_	1000 *g*	1020	_	/	_	300	_	_
H Li [163] (2020)	Electrostatic	Contact	Silicon bulk micromachining	0.7	_	0.4	1000 × 330	7.5	0	75	5 × $10^{6}$	MEMS relay
A S Bale [164] (2020)	Electrostatic	Capacitive	_	2.5	/	/	_	5	_	35	_	RF MEMS
